# Age and Muscle-Dependent Variations in Corticospinal Excitability during Standing Tasks

**DOI:** 10.1371/journal.pone.0110004

**Published:** 2014-10-13

**Authors:** Anthony Remaud, Martin Bilodeau, François Tremblay

**Affiliations:** 1 Bruyère Research Institute, Ottawa, Ontario, Canada; 2 School of Rehabilitation Sciences, University of Ottawa, Ottawa, Ontario, Canada; University of Bologna, Italy

## Abstract

In this study, we investigated how modulation in corticospinal excitability elicited in the context of standing tasks varies as a function of age and between muscles. Changes in motor evoked potentials (MEPs) recorded in *tibialis anterior* (TA) and *gastrocnemius lateralis* (GL) were monitored while participants (young, n = 10; seniors, n = 11) either quietly stood (QS) or performed a heel raise (HR) task. In the later condition, transcranial magnetic stimulation (TMS) pulses were delivered at three specific time points during the task: 1) 250 ms before the “go” cue (preparatory (PREP) phase), 2) 100 ms before the heel rise (anticipatory postural adjustment (APA) phase), and 3) 200 ms after heel rise (execution (EXEC) phase). In each task and each phase, variations in MEP characteristics were analysed for age and muscle-dependent effects. Variations in silent period (SP) duration were also examined for certain phases (APA and EXEC). Our analysis revealed no major difference during QS, as participants exhibited very similar patterns of modulation in both TA and GL, irrespective of their age group. During the HR task, young adults exhibited a differential modulation in the PREP phase with enhanced responses in TA relative to GL, which was not seen in seniors. Finally, besides differences in MEP latency, age had little influence on MEP modulation during the APA and EXEC phases, where amplitude was largely a function of background muscle activity associated with each phase (i.e., APA: TA; EXEC: GL). No age or muscle effects were detected for SP measurements. Overall, our results revealed no major differences between young adults and healthy seniors in the ability to modulate corticospinal facilitation destined to ankle muscles during standing tasks, with maybe the exception of the ability to prime muscle synergies in the preparatory phase of action.

## Introduction

In humans, transcranial magnetic stimulation (TMS) allows for non-invasive exploration of variations in corticospinal excitability associated with task performance. While much of the focus of previous work has been on the upper extremity with a large emphasis on hand muscles, relatively few studies have examined how the lower limb motor representation is subject to task-related modulation. This is due, in part, to the difficulty in eliciting reliable TMS responses in some leg muscles [1] and also to the fact that lower limbs are primarily involved in tasks dealing with posture and locomotion, which were traditionally thought to depend largely on brainstem and spinal cord circuitry [2]. Yet, evidence from both animal studies and neuropathological case studies in humans suggests a major role for cortical structures in influencing postural and locomotor neuronal networks [3]. Such a role in the case of the motor cortex has been highlighted in recent TMS studies examining modulation in corticospinal excitability in the context of postural and stepping tasks. For instance, Obata et al. [4] compared TMS responses elicited at different intensities in ankle flexor and extensor muscles during standing and sitting postures. Their results showed increased responsiveness to TMS in both flexors and extensors when standing as compared to sitting, suggesting postural-related enhancement in corticospinal excitability for the standing task. Similar task-related modulation were reported by MacKinnon et al. [5] during step initiation, where early facilitation were detected in motor evoked potentials (MEPs) in the *tibialis anterior* (TA) muscle 100 ms after the “go” cue. This early modulation suggested that corticospinal pathways could contribute to the generation of anticipatory postural adjustments (APAs) during step preparation. A corticospinal contribution to APAs generated in ankle muscles during postural tasks was also proposed by Petersen et al. [6]. These authors observed that MEPs elicited in the soleus muscle were facilitated up to 75 ms prior to self-induced perturbations, whereas such early modulation was not present when perturbations were unpredictable. In fact, one of the major roles ascribed to cortical structures in postural control is to adjust “central set” by priming brainstem and spinal cord circuits in advance to generate appropriate responses to a given context [3]. Collectively, these observations confirmed the critical role of descending projections arising from the motor cortex in modulating lower limb responses during postural and stepping tasks, especially with regard to the generation of APA.

Another factor known to influence postural control is age. With age, the ability to produce fast and efficient postural responses often declines, especially when balance conditions become more challenging [7]. Such a decline could be due to age-related changes at the peripheral and spinal levels (e.g., altered proprioception, sarcopenia) but it may also reflects, in part, alterations at the cortical level. For instance, older adults typically exhibit delayed APA activity in leg and trunk muscles during self-initiated movements of the upper extremity [8]–[10], which suggests deficit in central set and feedforward control. Impaired cortical functions are also evidenced in seniors through their difficulties in allocating attentional resources to maintain balance when a secondary cognitive task is performed while standing or walking, making them at risk for falls [11]. In line with this, neuroimaging studies have found that older adults tend to display more diffuse and widespread brain activity when performing motor actions [12], often extending to non-motor areas [13]; suggesting a loss of inhibition and/or the need for a greater recruitment at the cortical level to maintain performance (see [14] for a recent review). In parallel, age-related alterations are also found in TMS markers of corticospinal excitability and of intra-cortical inhibition [15], which can affect the ability of seniors to modulate the excitatory and inhibitory drive destined to limb muscles to meet task demands. In this regard, our own investigations in hand motor representation have shown that while most seniors retain the ability to produce task-related corticospinal facilitation, this facilitation is critically dependent on the ability to engage executive processes [16] and often occurs at the expense of a decrease in muscle selectivity [17]. However, there is still very limited information as to the effect of age on the ability to modulate corticospinal excitability in lower leg muscles in the context of standing tasks.

In the present study, we investigated posture-related variations in corticospinal excitability through responses elicited with TMS in lower limb muscles. Our goal was to first determine whether age would affect MEP modulations, and second, whether age would interact with muscle-dependent effects, when contrasting MEP responses obtained from ankle flexor and extensor muscles.

## Materials and Methods

### Ethics statement

The study procedures were approved by the Research Ethics Board at the Bruyère Research Institute, Ottawa, Ontario, Canada. Written informed consent was obtained prior to participation from all participants in accordance with the *Declaration of Helsinki*. All assessments were performed in a controlled laboratory environment. Each participant received a small honorarium for his or her participation.

### Participants

Ten healthy young adults (4 women and 6 men; 23.3±4.1 years, 171.9±11.2 cm and 70.8±13.9 kg) and 11 healthy seniors (6 women and 5 men; 66.6±5.5 years, 170.1±11.7 cm and 71.4±15.8 kg) were recruited for this study. All participants were recruited in the Ottawa-Gatineau area and all seniors were active community dwellers. Participants completed a medical questionnaire to ensure that there was no contra-indication to TMS (e.g., epilepsy, use of cochlear implants and/or a pace-maker) and no antecedents of conditions likely to affect their performance in the tests (e.g., arthritis, lower-limb injury in the six months prior to data collection).

### Electromyographic recordings and TMS procedures

Surface electromyographic (EMG) activity was recorded using auto-adhesive surface electrodes (Ag/AgCl, Kendall Medi-Trace 230) placed over the TA and *gastrocnemius lateralis* (GL) muscles of the right lower leg. Electrodes were placed on the muscles according to SENIAM recommendations [18], with the inter-electrode axis aligned with the assumed direction of muscle fibres. EMG signals were amplified (AB-621G Bioelectric amplifier, Nihon-Kohden Corp., Irvine, CA, USA), digitized at a rate of 2 kHz (BNC-2090, National Instrument Corp., Austin, TX, USA) and further relayed to a laboratory computer running custom software to control acquisition.

Before formal testing, participants underwent TMS to determine the optimal site on the scalp to evoke MEPs in the leg muscles (i.e., TA and GL) and then to establish the resting motor threshold (rMT). These parameters were determined with participants comfortably seated in a recording chair. TMS was produced via a Magstim 200 (Magstim Corp., Whitland, UK) connected to a double cone coil (MagStim P/N: 9902, 96-mm loops). For the hotspot determination, the coil was displaced in 1-cm steps starting from the vertex in the anterior direction while stimulating at high intensity (50% stimulator output) until MEPs ≥25 µV could be reliably evoked either in TA or GL. However, for many participants, MEPs were easier to evoke in the TA and thus the rMT was based mainly on responses evoked in this muscle. Once identified, the position of the coil was marked by placing red stickers on the scalp. With the coil held in place by one of the experimenter, the rMT was determined using the Motor Threshold Assessment Tool software (MTAT 2.0, freeware available at: http://clinicalresearcher.org/software.html) developed by Borckardt et al. [19]. The software allows for fast estimation of motor threshold through the maximum-likelihood strategy based on the PEST (Parameter Estimation by Sequential Testing) algorithm [20]. This method yielded rMTs in the young and senior groups (38±5%; 48% ±10%, respectively) comparable in range to those reported in our previous TMS investigation in the lower limb motor representation with other threshold estimation methods [21], [22].

### Corticospinal excitability during standing tasks

Corticospinal excitability was tested under similar conditions for all tests. Testing was performed with participants standing in front of a table adjusted at waist level for safety reasons and wearing only socks. The coil was placed on the top of the participant’s head and held firmly in place by one of the investigator (FT), who stood on a stool just behind the participant. The position of the coil was re-assessed frequently during trials to ensure consistent positioning. The TMS intensity was set at 110% of the rMT for all experimental trials. In all participants, corticospinal excitability was first examined during quiet standing (QS) and then during the heel-raise (HR) task. For testing during QS, participants were simply asked to stand as still as possible, while TMS pulses were delivered at random intervals (5–10 s between pulses) until 10 MEPs were recorded. The MEPs traces were saved and stored for off-line analysis.

**Figure 1:**
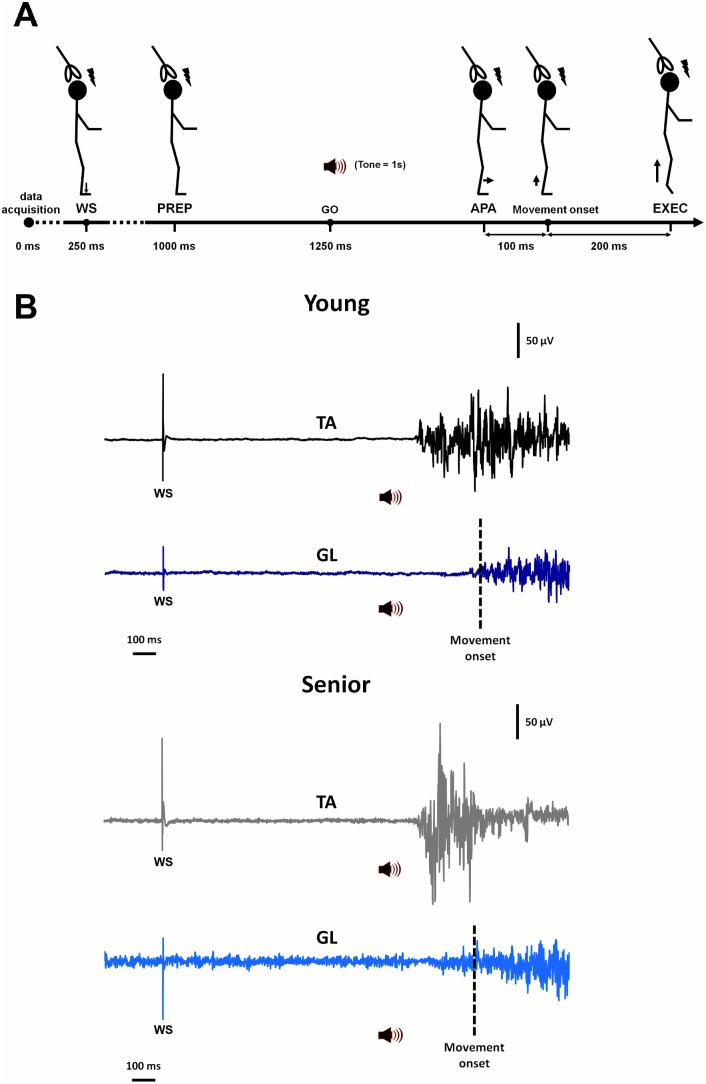
Schematic representation of the experimental protocol and examples of muscle activity elicited in *tibialis anterior* (TA) *gastrocnemius lateralis (GL)* during performance of the heel raise (HR) task in participants of each age group. Responses to transcranial magnetic stimulation were recorded under two task conditions, quiet standing (QS, not shown here) and HR, with the latter task being subdivided in three phases. As shown in A, each HR trial (3000 ms epoch) included a warning signal (WS) in the form of an electrical pulse delivered at the dorsum of the foot at 250 ms (thin arrow symbol), followed by a “go” cue at 1250 ms in the form of an auditory tone lasting 1 s. Participants were instructed to synchronize the heel raising action with the tone. In the electromyographic traces shown in B, the onset of the anticipatory postural adjustment (APA) elicited in the TA in preparation for the upcoming action along with the actual onset of the heel raising action in the GL are clearly evident in the two participants, young and senior. Such recordings, obtained prior to TMS applications, were used to individually adjust stimulation delays during performance of the HR task. As illustrated in A (lightning symbol), the earlier time delay for TMS delivery was in the preparatory (PREP) phase at 1000 ms (i.e., 250 ms before the go cue), when participants prepare to the upcoming action. The second delay for TMS was set at 100 ms before movement onset in the APA phase, where participants moved their body forward to anticipate the heel raising action. Finally, TMS was delivered 200 ms after movement onset during actual execution of the heel raising action (EXEC).

Prior to testing with the HR task, participants performed a series of trials (n = 8) with the coil in place but with no stimulation being delivered. These pre-TMS trials served to familiarize participants with the task and to determine the onset of the APA and the movement onset. Each HR trial consisted of 3000 ms epoch. The first event consisted in a warning cue at 250 ms in the form of a weak electrical pulse (200 µs, 120% sensory threshold) delivered at the dorsal aspect of the foot by means of surface electrodes connected to a S88 stimulator (Grass Corp., Warwick, RI, USA). This warning cue was followed 1000 ms later by the “go” cue at 1250 ms, which consisted in a 500 Hz auditory tone lasting 1000 ms generated by the computer. The auditory tone was delivered through headphones so that participants could focus on the task and ignore other source of noise, notably the clicking noise produced by the coil in trials with TMS. In response to the “go” cue, participants were instructed to slowly rise on their tiptoes synchronously with the tone. The 1000 ms duration for the task was chosen after pilot testing demonstrated that this speed was comfortable for seniors and did not compromise coil stabilization. From visual inspection of TA and GL EMG traces, a mean APA and movement onset time was determined for each participant (onset determined as the time when EMG activity increased to a value at least twice that of the noise background level). The mean movement onset time was then used to adjust TMS delivery times for each participant. The first delay was set at 250 ms before the “go” cue (preparatory phase: PREP), in the foreperiod between the warning signal and the “go” signal. The second was set at 100 ms before movement onset in the phase where anticipatory activity is generated in the TA (APA phase) to move the centre of mass forward. The third delay was set at 200 ms after the movement onset during the actual heel raise execution (EXEC phase) when the GL was mostly active. After adjusting timing delays for TMS, variations in corticospinal excitability were measured in each participant in a series of 30 consecutive trials where TMS pulses were delivered according to a pre-established random sequence so that 10 MEPs could be recorded for each of the three phases (i.e., PREP, APA and EXEC). Every 10 trials, participants were given a 2-min rest period to avoid fatigue. A schematic illustration of the experimental paradigm is provided in Figure 1 (A) along with examples of EMG traces obtained during performance of the HR task (B).

After completion of the standing trials with TMS, participants were asked to perform three static contractions (3 s each) with the ankle either maximally dorsi-flexed or plantar-flexed (i.e., standing on their heels or their tiptoes). These static contractions served as a reference index to estimate the level of background EMG activity produced in the target muscles (TA and GL) during the standing tasks.

### Data analysis

All MEP data were analyzed off-line by the same investigator (AR). First MEPs recorded in each condition were overlaid and then averaged to get a mean amplitude (peak-to-peak) and latency for each participant. The duration of the silent period (SP) was also measured in the two phases of the HR tasks (i.e., APA and EXEC) where background activity was sufficiently high and sustained to detect a decline in activity. To reduce errors associated with determination of the SP onset, the duration was determined as the time interval from the TMS pulse to the first sign of sustained (>10 ms) recovery in EMG activity (e.g. [23]). Finally, since MEP facilitation can vary depending on the level of contraction, EMG activity produced in TA and GL during the standing tasks was quantified with respect to the reference contractions. For this quantification, the EMG activity generated in TA and GL during the 3 s static contractions was first rectified and then averaged across a 500 ms window (2000–2500 ms) to get a maximal reference contraction (MRC). Then, the mean rectified EMG activity produced in TA and GL during performance of the QS and HR tasks in the 50 ms window preceding the TMS pulse was expressed as a percentage of their respective MRC to get an estimate of the level of task-related activity.

### Statistical analysis

Since our goal was to examine modulations in corticospinal excitability within each task or phase with regard specifically to “age” and “muscle” effects, separate two-way mixed-design ANOVAs (age [young adults, older adults] × muscle [TA, GL]) were performed on MEP amplitude and latency data for each task/phase (QS, PREP, APA, EXEC). Similar two-way mixed-design ANOVAs were also performed on SP duration but only for the APA and EXEC phases of the HR task (i.e., the two phases where SP could be easily detected). Note that since MEP amplitudes were not normally distributed, individual mean values were transformed into natural logarithms, as suggested by Nielsen [24]. Following this transformation, MEP amplitudes were normally distributed (Kolmogorov-Smirnov test *p*>0.05). Latency and SP duration values were normally distributed and required no transformation. In addition, to test whether aging influenced pre-stimulus background EMG activity, separate two-way mixed-design ANOVAs (age × muscle) were also performed on EMG levels computed for each task/phase (QS, PREP, APA, EXEC). Pearson’s correlations were used to examine association between task-related variations in MEP amplitude and background EMG activity in TA and GL. When the sphericity assumption in ANOVAs was violated (Mauchly’s test), a Geisser/Greenhouse correction was used. If relevant, *post hoc* tests were performed by means of the Newman-Keuls procedures. Statistical significance was set at *p*<0.05. All results are reported as mean ± standard deviation.

## Results

### General observations

All participants completed the testing without difficulty or loss of balance during the standing tasks. The average stimulation intensity to test excitability was respectively 42% (±6%) and 53% (±11%) in the young and senior groups, reflecting the difference in rMT between the two groups.

### Corticospinal excitability during QS

**Figure 2:**
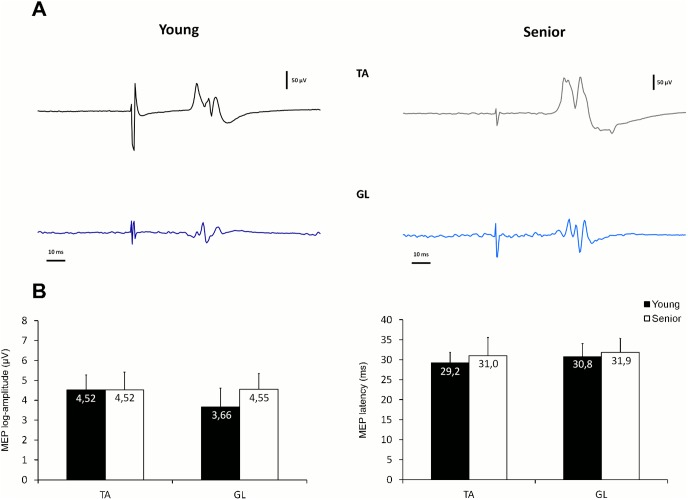
Characteristics of motor evoked potentials (MEP) recorded during quiet standing. **A.** Examples of individual MEP traces recorded in TA and GL during quiet standing, in a young and a senior participant. **B.** Mean MEP log-amplitude and latency computed in each age group during quiet standing. Note that no main effect or interaction was detected. Abbreviations as in Figure 1.

In terms of background EMG activity, as expected, the ANOVA revealed a substantial “muscle” effect (F_1,19_ = 14.2, p = 0.001, η^2^_p_ = 0.43) while no “age” effect was detected. The large “muscle” effect reflected the fact that the GL exhibited, on average, higher tonic activity during QS than the TA in both age groups (data pooled across age groups: 7% vs. 2%, for GL and TA respectively). As shown in Figure 2 (A and B), this difference in background EMG activity between TA and GL was not reflected in MEP amplitude, as no “muscle” effect was detected in the ANOVA (F_1,19_ = 2.5, p = 0.128, η^2^_p_ = 0.12). No main effect or interaction was detected with regard to the influence of “age” on MEP amplitude, although MEPs in GL of young participants tended to be of smaller size than those in seniors. Also evident in Figure 2B is the fact that measures of MEP latency were very similar between TA and GL and were little affected by age. Correlations between MEP amplitude and background EMG levels revealed no significant association either in TA or GL (r<0.3, p>0.2).

### Corticospinal excitability during HR

The mean APA and movement onsets computed from trials without TMS were very similar in the two age groups. In the young group, the averaged APA onset time, as reflected in TA activation, was 1351±69.5 ms, whereas it was 1360±29.2 ms in the senior group. Movement onset time, as determined from GL onset activation, was respectively, 1621±110.2 and 1583.6±69.6 ms, in young and senior adults. Comparisons with t-tests for independent samples confirmed that no significant difference existed between the two age groups both for APA and movement onset times (t_19_<0.94, p>0.35).

#### PREP phase

**Figure 3:**
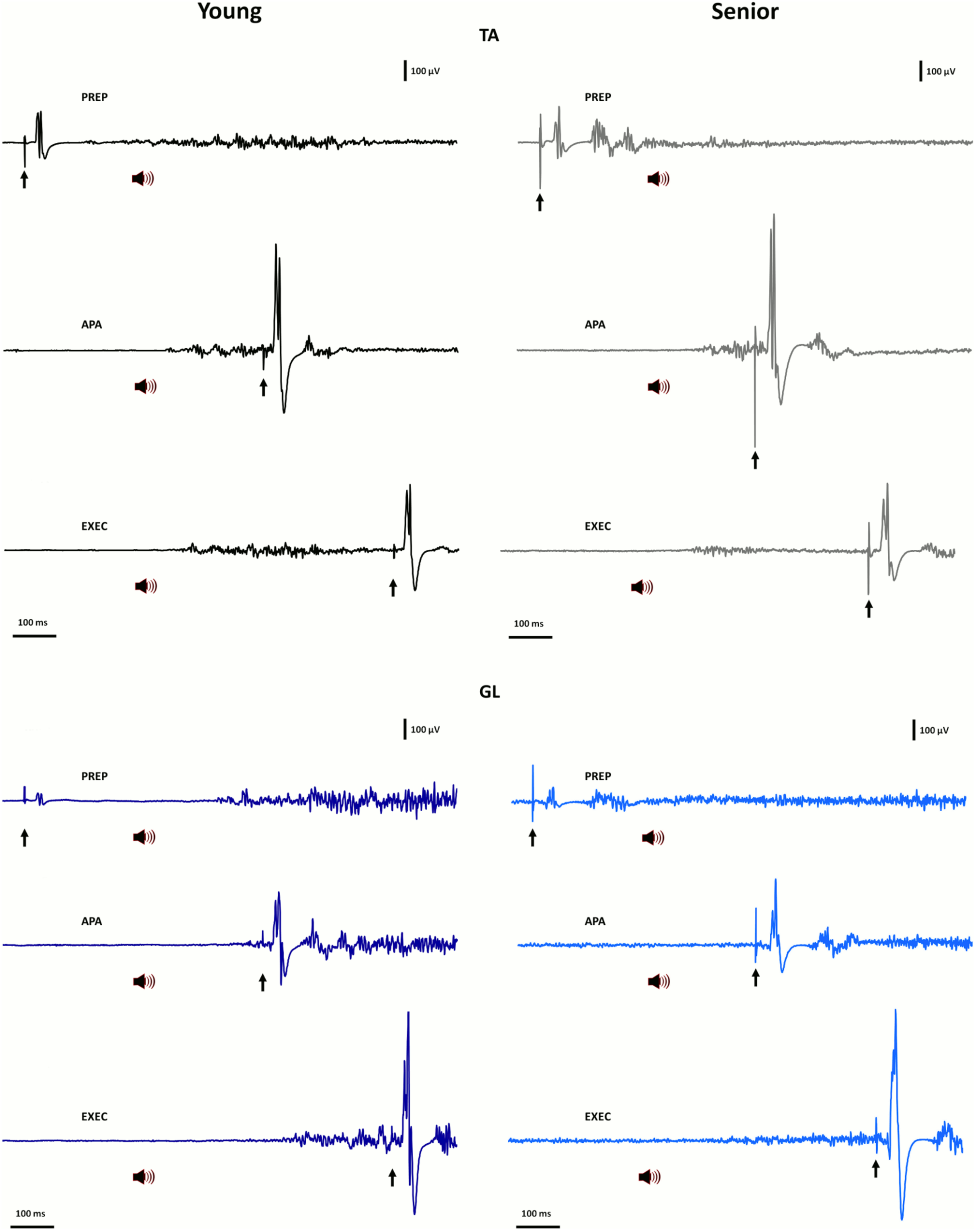
Examples of background electromyographic activity and of MEP recorded in TA and GL in a young and a senior participant during performance of the heel raise task. In each participant and for each muscle, the activity and MEP responses are given for each time delay (indicated by arrows) and their corresponding phase: PREP phase (1000 ms, 250 ms before the “go” cue); APA phase (100 ms before movement onset) and EXEC phase (200 ms after movement onset) respectively for the young and senior participants. The “go” cue is indicated by a speaker symbol. Abbreviations as in Figures 1 and 2.

**Figure 4:**
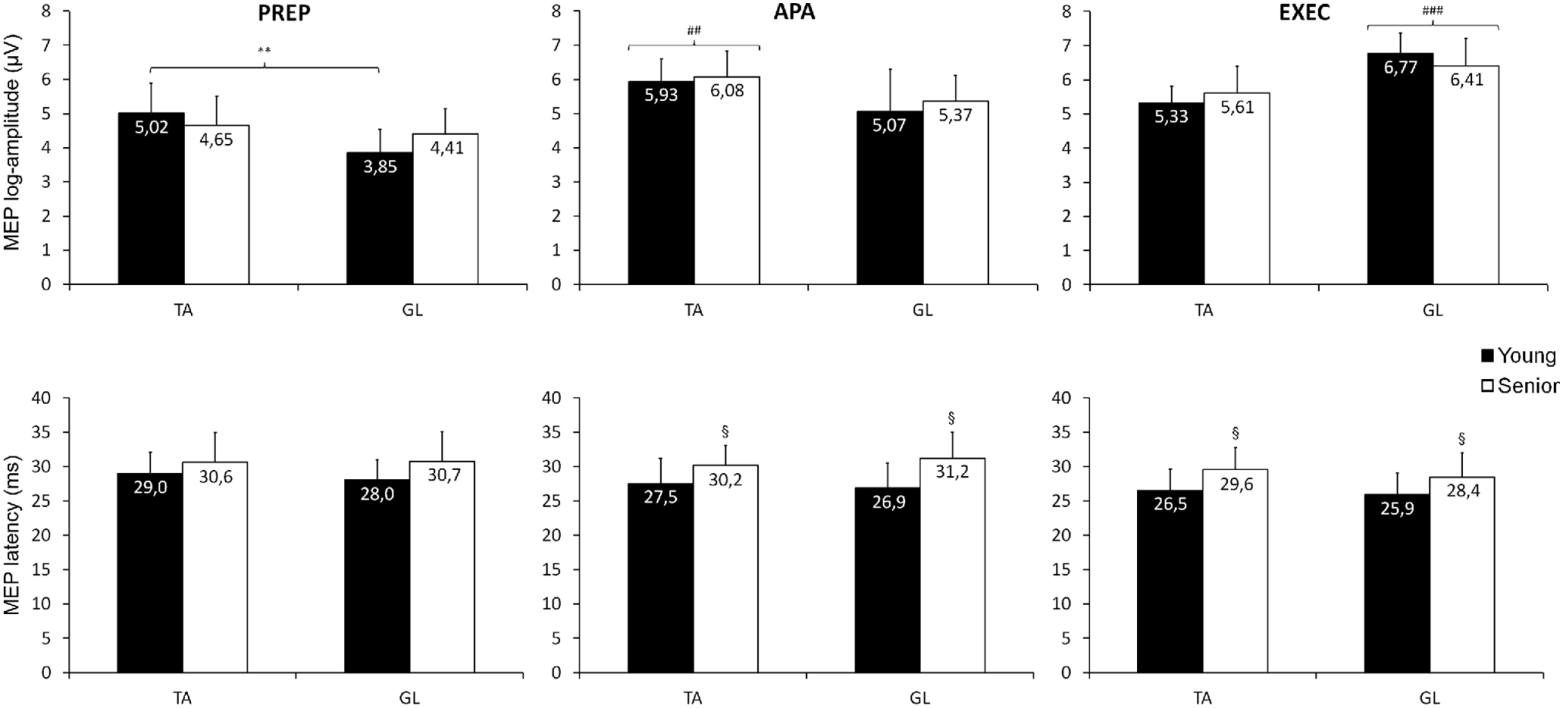
Mean MEP log-amplitude (upper panel) and latency (lower panel) computed in TA and GL in the two groups at each time delay/phase of the heel raising task. Symbols indicated significant main effects or interactions. Note that in the PREP phase, a significant group×muscle interaction (indicated by **, p<0.01) was found reflecting the difference in MEP amplitude between TA and GL. In the APA and EXEC phases, a significant main effect of muscle (indicated by ## and ###, p<0.01 and p<0.001 respectively) was found reflecting larger MEP amplitude in the TA and in the GL during their respective phase. A main effect of group (indicated by §, p<0.05) was found for MEP latency reflecting delayed latency in seniors in the APA and EXEC phases. Abbreviations as in Figures 1, 2 and 3.

Much like the QS task before, analysis of variations in background EMG activity in the PREP phase also revealed a large “muscle” effect (F_1,19_ = 13.1, p = 0.002, η^2^_p_ = 0.41), owing to the greater activity observed in GL (8%) than in TA (3%) in the two age groups. In contrast to the QS, however, analysis of variations in MEP amplitude revealed a significant “age×muscle” interaction (F_1,19_ = 5.6, p = 0.029, η^2^_p_ = 0.23). This interaction reflected the fact that young participants exhibited, on average, greater facilitation in TA than in GL in the PREP phase, whereas no such difference was found in the senior group. An example of such differential modulation is given in Figure 3 showing MEP modulation recorded at the different phases in a young and a senior participant. The differential modulation between TA and GL MEP amplitude is evident in the young subject when compared to the senior participant. The “age×muscle” interaction found for MEP amplitude in the PREP phase is also evident from inspection of Figure 4. Also shown in Figure 4, is the fact that no main “age” or “muscle” effect was detected in MEP latency (F_1,19_ = 2.3, p = 0.148, η^2^_p_ = 0.11 and F_1,19_ = 0.3, p = 0.597, η^2^_p_ = 0.01 respectively). Similar to the QS task, no significant correlation could be found between background EMG level and MEP amplitude recorded in TA and GL (r<0.28, p>0.22) during the PREP phase.

#### APA phase

During the APA phase, a main “age” effect was found when comparing background EMG activity (F_1,19_ = 5.6, p = 0.029, η^2^_p_ = 0.23) between groups, with no “muscle” effect (F = 0.9, p = 0.355, η^2^_p_ = 0.05). The “age” effect reflected the fact that seniors exhibited on average greater overall muscle activity in this phase than their younger counterparts (TA and GL activity pooled: 19% vs. 8%, respectively). In contrast, measures of MEP amplitude showed only the expected “muscle” effect (F_1,19_ = 11.0, p = 0.004, η^2^_p_ = 0.37) owing to the larger MEP size observed in TA as compared to GL (p = 0.004). This difference is clearly seen in the individual traces shown in Figure 3 (middle traces in upper and lower panels) and in the corresponding mean values illustrated in Figure 4. Consistent with this “muscle” effect, variations in background EMG activity in TA were positively correlated with MEP amplitude (r = 0.53, p = 0.014), whereas no such association was found for GL (r = 0.28, p = 0.22). A main “age” effect was again found for variations in MEP latency (F_1,19_ = 6.1, p = 0.023, η^2^_p_ = 0.24), with pooled latencies in TA and GL being on average 3.5 ms longer in seniors than in young participants (Figure 4). Further analysis of variations in SP duration did not reveal any main effect or interaction (p>0.05).

#### EXEC phase

As expected, a main “muscle” effect was found for background muscle activity (F_1,19_ = 38.8, p<0.001, η^2^_p_ = 0.67) while no “age” effect was detected. In fact, in this phase, the overall level of background EMG activity was considerably higher in GL (54%) than in TA (10%). Accordingly, a substantial “muscle” effect (F_1,19_ = 52.9, p<0.001, η^2^_p_ = 0.74) was also found for MEP amplitude, which reflected the higher facilitation elicited in the GL as compared to the TA (p<0.001). This difference in facilitation can be easily appreciated by inspecting Figure 3 (lower trace, upper and lower panels) and Figure 4. Variations in MEP amplitude in the EXEC phase in GL were also strongly correlated with corresponding variations in EMG activity (r = 0.57, p = 0.007), while no such correlation was found for TA (r = 0.14, p = 0.50). As found for the APA phase, a main “age” effect was found for variations in MEP latency (F_1,19_ = 4.4, p = 0.049, η^2^_p_ = 0.19) owing to the longer latencies (2.8 ms on average) observed in seniors than in young participants (figure 4, bottom right panel). No main effect nor interactions were found for variations in SP duration (p>0.05).

## Discussion

In the present study, we investigated age and muscle-dependent variations in corticospinal excitability of ankle muscles during standing tasks. Three main findings emerged from our observations. First, TMS responses were very similar in TA and GL, irrespective of the age group for the QS task. Second, during the HR task, young adults exhibited a differential modulation between TA and GL in the foreperiod (PREP phase), which was not seen in the senior group. Finally, other than differences in MEP latency, age had little influence on the modulation observed during the APA and EXEC phases, where MEP amplitude in TA and GL was strongly influenced by the respective background muscle activity associated with each phase (i.e., APA: TA; EXEC: GL).

### Modulation during QS

As stated above, both young and senior adults exhibited similar patterns of modulation when MEPs were elicited during QS. In addition, no major difference was found between TA and GL in terms of MEP amplitude. Our observations with regard to age are somewhat in contrast with those of Baudry et al. [25], who recently reported an increase in corticospinal excitability during upright stance in elderly when compared to young adults. Methodological differences could explain this apparent discrepancy as Baudry et al. [25] examined corticospinal excitability across a wide range of intensities and used the soleus as the target muscle, whereas a single test intensity was used in the present study with the GL as the target muscle. Still, two factors might have contributed to mitigate age differences in our study. First, as reported in other studies (e.g., see [26]), senior adults tended to show higher rMTs than young adults, and thus, higher intensities were used to test excitability in this group, a finding that might have contributed to reduce differences in MEP amplitude. On the other hand, elevation of rMT in seniors are not always indicative of decreased cortical excitability and may reflect changes in the skull-cortex distance (e.g. [27]) or even changes in the white matter integrity [28]. The second factor is the fact that our group of seniors was relatively “young” and active (see Limitations below), which could have attenuated differences related to age. Indeed, there is evidence from posturographic studies [29], [30] that balance control in healthy seniors is relatively unaffected when tested under unchallenging conditions, such as in the present study.

With regards to potential muscle-dependent effect, we did not observe larger MEP responses in the GL in spite of the greater tonic background activity recorded in this muscle during QS. This observation might be explained by the fact that MEPs in GL were generally smaller in size than those evoked in TA, which could have contributed to attenuate differences in amplitude between the two muscles. In fact, TMS responses to cortical stimulation are typically harder to elicit in ankle extensors than in flexors, owing to differences in the strength of corticospinal projections between the two muscle groups [1], [31]. While weaker descending facilitation to GL is possible, there is evidence that both ankle flexors and extensors undergo similar cortical modulation during postural activity. Indeed, much like in the present study, Obata et al. [4] observed no major difference between TA and *soleus* muscles in terms of responsiveness when comparing TMS responses obtained in standing vs. sitting posture over a wide range of intensities. Thus, our observations suggest no major alterations in the ability to modulate corticospinal facilitation destined to ankle muscles during QS in active healthy seniors.

### Modulation during the HR task

During the HR tasks, both senior and young adults exhibited similar APA onset and movement onset times, indicating that the two groups were able to synchronize their performance with the auditory signal. According to several reports (e.g., see [32], [33] postural activity elicited in leg muscles during standing tasks tends to increase with age. This increase in postural activity has been ascribed to compensations linked with age-related changes in the ability to generate muscle tension and to produce torque at the ankle [32]. While we did observe a tendency for higher level of background activity in the senior group, the difference between groups was significant only for the APA phase, where seniors exhibited increased activity in the TA. This increased activity in the TA might be linked to change in motor preparation in the senior groups, as discussed below.

Performance of the HR task included a foreperiod between the warning signal and the “go” signal, during which participants prepared their heel raising action. Interestingly, it is in this foreperiod (i.e., PREP phase) that young adults displayed significantly enhanced TMS responses in the TA when compared to GL. In previous TMS studies, modulation of MEP responses in the period between a warning and an imperative stimulus has been shown to vary depending on the length and variability of the foreperiod. When the foreperiod is short (500–1000 ms) and constant across trials, as in the present study, MEPs in the agonist muscle tend to be suppressed [34], likely reflecting the importance of motor cortical inhibition in preventing premature responses. As stressed by Sinclair and Hammond [35], such depression is thought to be the result of a competing process whereby excitatory and inhibitory influences are balanced to optimize motor cortical excitability in preparation for action. The actual differential modulation observed between TA and GL in the PREP phase is likely a reflection of this competing process in the context of the task, where facilitation of the TA prepared for the upcoming APA, while MEPs in GL (agonist muscle) were either unchanged or depressed to prevent premature responding. While we cannot rule out the possibility that the differential modulation might have been influenced by the differences in TMS responsiveness between TA and GL (see above discussion), such influence seems marginal for several reasons. First, the differential modulation was seen only in one group and notably absent in the senior group. Second, no differential modulation was found during QS, as discussed above. Finally, both TA and GL showed the expected activity-dependent modulation in the phases where the two muscles were mostly active (APA and EXEC), indicating that TMS responses in each muscle were sensitive in reflecting task-related corticospinal influences.

The reason as to why the differential modulation was not seen in the senior group might be related to age-dependent changes in preparatory processes. Older adults typically exhibit slower response times and this delay has been ascribed in part to inefficiency in motor preparation processes, as evidenced by alterations found in readiness potentials in EEG studies (e.g., attenuated contingent negative variation amplitude [36]). In TMS studies, evidence of a decline in motor preparation with age has been linked with reports of reduced intra-cortical inhibition in older adults [37], [38] since, as stressed before, motor cortical inhibition seems to be a critical component of the preparation process to prevent unwanted responses. Changes in motor preparatory processes with age can also vary depending on task conditions. For instance, Levin et al. [39] showed that older adults exhibited a readiness strategy to speed up their response in a simple reaction time task, whereby they increased the excitability of corticospinal projections to the active hand while suppressing the excitability of those of the resting hand. Such differential modulation was not seen in younger participants and presumably reflected an optimized preparatory strategy to ensure fast and accurate responses. Evidently, the context of our HR task was quite different, where the focus was on movement execution in pace with the tone and not speed or accuracy.

As stated before, in the later two phases of the HR task, MEP amplitude modulation in TA and GL was driven mainly by phase-specific variations in the level of muscle activity (TA for APA phase, GL for EXEC phase). This was clearly evident by the strong correlation found between background EMG level elicited in each phase and MEP amplitude. In these two phases, the increased responsiveness to TMS was expected as increase in the voluntary drive results in larger descending volleys coupled with enhanced spinal excitability arising from peripheral afferent feedback from the contracting muscle [40]. The observation that age had no effect on MEP amplitude during the APA and EXEC phases might be taken as evidence that mechanisms modulating cortical and spinal excitability during the HR were preserved in the senior group. On the other hand, senior participants did exhibit signs of impaired facilitation in the form of a delayed MEP onset latency. Indeed, beyond increased amplitude, the other critical manifestation of contraction-induced MEP facilitation is a reduction in onset latency, reflecting improved temporal summation as the threshold to activate motoneurones is lowered at the spinal level [41]. In this respect, the delayed latencies exhibited by the seniors when TMS was delivered in the APA and EXEC phases suggest impaired temporal summation likely related to increased disparities in fast conducting corticospinal fibers with age leading to slower activation time. It is also possible that peripheral changes in nerve conduction might have contributed in seniors, although this contribution seems negligible given that age differences in latency were significant only for the APA and EXEC phases, i.e., during contraction-induced facilitation.

Finally, in agreement with our previous studies examining task-related modulation in upper extremity muscles [16], [42], variations in the SP duration measured in the APA and EXEC phases were not influenced by age. As the ability to produce smooth and coordinated movement tends to decline with age [43], some TMS research looked at the SP, a marker of motor cortical inhibition [44], to investigate potential age-related changes in motor coordination. These investigations [45], [46] showed that older adults exhibited shorter SP duration (i.e., less cortical inhibition) than young adults during difficult motor coordination tasks. These authors also noticed that a short SP duration was associated with poor coordination in older adults, thus suggesting a direct link between motor coordination and the ability to control cortical inhibition. In the present study, it is likely that both the healthy status of our senior participants and the fact the postural task was not really challenging in terms of motor coordination could account for the lack of an age effect on SP duration.

### Limitations

There are two main limitations in the present study. First, as stressed before, the fact that our group of seniors was relatively “young” (66.6±5.5 years) and healthy could have masked potential age differences in corticospinal excitability during the tasks, limiting our observations to an active healthy senior population. Second, the fact that the HR task was controlled so that the speed of execution was constrained in time may also have contributed to mask differences due to age. As stated before, this aspect of the experimental protocol was introduced both for safety concerns and to control stimulation conditions (i.e., to avoid coil displacement).

## Supporting Information

File S1 (XLS)
